# Ultra-rapid rollout vaccination with BNT162b2 to reduce SARS-CoV-2 infections in the general population

**DOI:** 10.1016/j.isci.2022.105380

**Published:** 2022-11-07

**Authors:** Lena Tschiderer, Lisa Seekircher, Lukas Richter, Dorothee von Laer, Cornelia Lass-Flörl, Lukas Forer, Sebastian Schönherr, Florian Krammer, Sabine Embacher-Aichhorn, Herbert Tilg, Günter Weiss, Franz Allerberger, Peter Willeit

**Affiliations:** 1 Institute of Health Economics, Medical University of Innsbruck, Anichstraße 35, 6020 Innsbruck, Austria; 2 Institute of Infectious Disease Epidemiology, Austrian Agency for Health and Food Safety, 1220 Vienna, Austria; 3 Institute of Virology, Medical University of Innsbruck, 6020 Innsbruck, Austria; 4 Department of Hygiene and Medical Microbiology, Medical University of Innsbruck, 6020 Innsbruck, Austria; 5 Institute of Genetic Epidemiology, Medical University of Innsbruck, 6020 Innsbruck, Austria; 6 Department of Microbiology and Department of Pathology, Molecular and Cell Based Medicine, Icahn School of Medicine at Mount Sinai, New York, NY 10029-5674, USA; 7 Clinical Trial Center, Medical University of Innsbruck, 6020 Innsbruck, Austria; 8 Department of Internal Medicine I, Gastroenterology, Hepatology, Endocrinology and Metabolism, Medical University of Innsbruck, 6020 Innsbruck, Austria; 9 Department of Internal Medicine II, Infectious Diseases, Immunology, Pneumology and Rheumatology, Medical University of Innsbruck, 6020 Innsbruck, Austria; 10 Department of Public Health and Primary Care, University of Cambridge, Cambridge CB1 8RN, UK

**Keywords:** Health sciences, Population, Immunology

## Abstract

This study aimed to determine the impact of ultra-rapid rollout vaccination on incidence of SARS-CoV-2 infection. Vaccination with BNT162b2 was provided to 66.9% of eligible residents of the Schwaz district in Tyrol, Austria, within six days per dose (first dose: 11–16 March 2021, second dose: 8–13 April 2021). Of 11,955 individuals enrolled at nine vaccination centers (median age 44.6 years; 51.3% female), 71 had incident SARS-CoV-2 over a six-month follow-up. Incidence rates per 100,000 person-weeks were 92.3 (95% confidence interval [CI]: 70.8–120.2) at weeks 1–5 and 6.4 (3.9–10.4) at ≥6 weeks after dose 1. In these two periods, effectiveness of the vaccination campaign to reduce incident SARS-CoV-2 was 58.6% (50.8%–65.2%) and 91.1% (89.6%–92.3%) in study participants and 28.3% (23.1%–33.0%) and 64.0% (61.7%–66.1%) in the Schwaz district, compared with districts with slower vaccination rollout. Therefore, the vaccination campaign in the Schwaz district illustrates the impact of accelerated vaccination rollout in controlling the pandemic.

## Highlights

• This study accompanied an ultra-rapid rollout vaccination campaign in Austria
• 66.9% of eligible residents of the Schwaz district received BNT162b2 within 6 days
• Over six months, SARS-CoV-2 incidence rate was 22.8 per 100.000 person-weeks
• Effectiveness of the vaccination campaign was 91.1% at ≥6 weeks after the 1^st^ dose

## Graphical abstract

**Figure:**
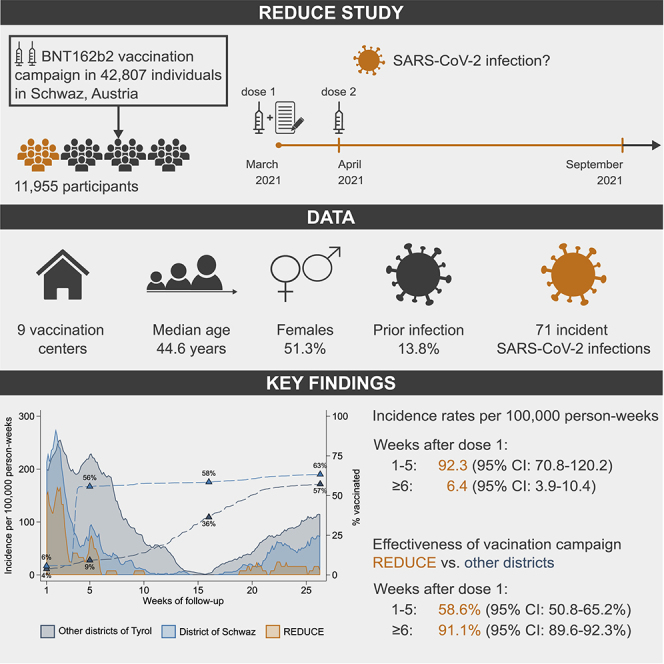

## Introduction

Vaccination against the severe acute respiratory coronavirus 2 (SARS-CoV-2) is the cornerstone in fighting the pandemic, with over 12 billion doses administered worldwide so far.^1^ In 2020, randomized controlled trials of SARS-CoV-2 vaccines provided promising results with respect to vaccine efficacy,^2^^,^^3^^,^^4^ and mass vaccination programs have been initiated around the world. Vaccination rollout has been observed particularly closely in Israel because the country achieved a vaccine coverage of 61% within four months and could therefore inform and provide useful guidance to other countries.^5^^,^^6^

Vaccination rollout in Austria started on 27 December 2020 in a staggered manner prioritizing individuals considered to be at higher risk for severe disease or those working in the health care sector. By that time, Austria had already registered over 350,000 cases of SARS-CoV-2 infections and more than 7,300 deaths caused by coronavirus disease 19 (COVID-19).^7^ In March 2021, the European Union allocated a batch of 100,000 additional doses of the BNT162b2 mRNA vaccine to the Republic of Austria.^8^ This was to provide ultra-rapid vaccination to the Austrian district of Schwaz in the Federal State of Tyrol to counteract particularly high numbers of SARS-CoV-2 infection. At that time, the district harbored the largest number of infections with the Beta variant (B.1.351) outside South Africa^9^ and the Alpha + E484K subvariant (B.1.1.7 + E484K), which were both deemed to partially escape vaccine-induced immune control.^10^^,^^11^ The vaccination campaign in the district of Schwaz was planned and set up within only one week and involved adverts and press releases to enhance public awareness, an online registration system, and vaccination centers in public buildings placed strategically across the district to enhance participation of the general public.

The vaccination campaign in the district of Schwaz provided a unique setting to study the impact of ultra-rapid rollout vaccination on incidence of SARS-CoV-2 infection in the general population. We therefore conducted a prospective cohort study—the REDUCE study—that accompanied the campaign and aimed to investigate its impact on incidence of SARS-CoV-2 infection overall and according to SARS-CoV-2 variants.

## Results

### Participants

**Figure 1:**
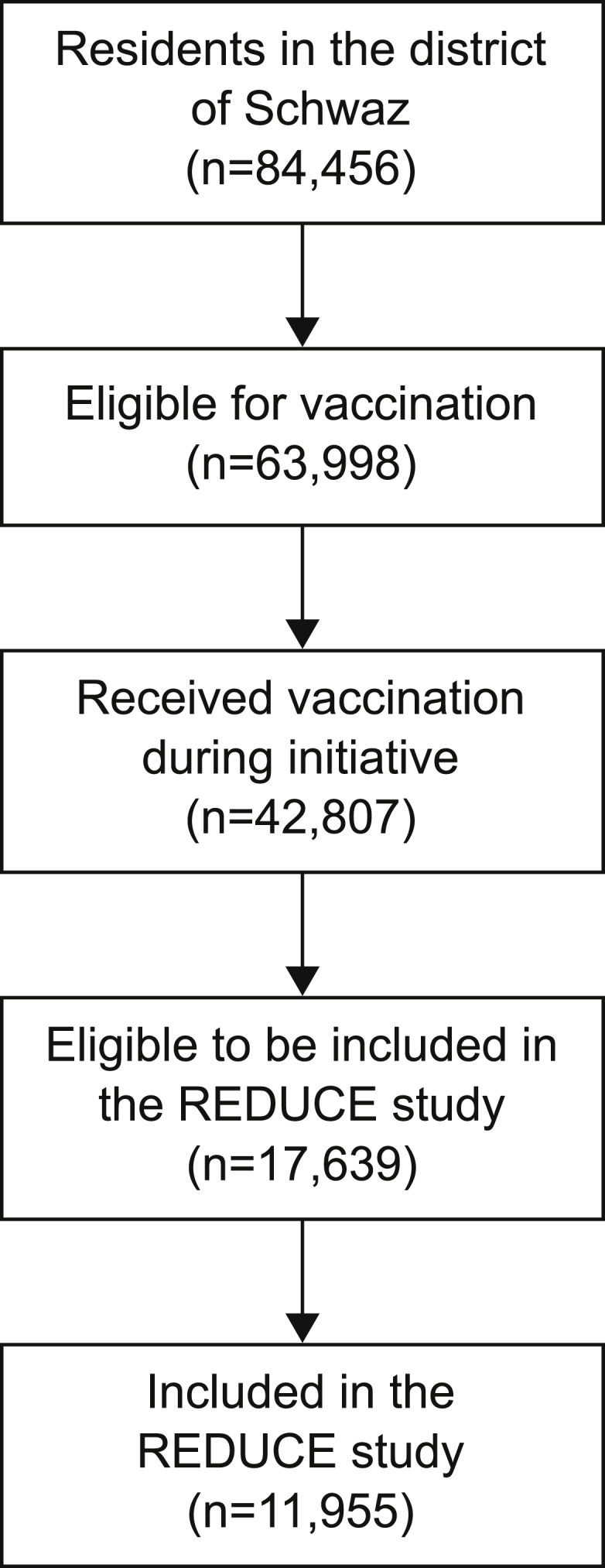
Participant flow chart

The baseline characteristics of the 11,955 participants enrolled in the REDUCE study (see Figure 1 for participant flow chart) are shown in Table 1. The median age was 44.6 years (interquartile range [IQR] 32.2–55.8) and 51.3% were female. Participants lived in households with a median of 3 persons (IQR 2–4) and had a mean body mass index of 25.4 kg/m^2^ (standard deviation 4.6); 26.3% of the participants were current smokers, 72.5% were currently employed, and 51.0% had a high education defined as a vocational school, A-levels, or university degree; 13.8% reported a prior SARS-CoV-2 infection, which was symptomatic in 75.2% and occurred in a median of 4.0 months before the study (IQR 3.4–4.5). Pre-existing diseases were rather infrequent, but 11.4% reported to have a history of cardiovascular disease and 4.2% reported to have chronic lung disease. Only 62 participants (0.5%) deviated from the vaccination regimen recommended by the national vaccination committee. These participants differed from the remaining study population in terms of occupational status (p = 0.048), history of renal disease (p = 0.042), history of chronic lung disease (p = 0.047), and intake of immunosuppressants (p = 0.042), but not in terms of other baseline characteristics (see Table S1). The median time between receipt of the first dose and the second dose was 28 days (5^th^–95^th^ percentile 27–28).

**Table 1:** Baseline characteristics of participants of the REDUCE study

Characteristic	Total no.	No. (%), mean ± SD or median (IQR)
Age, years	11,955	44.6 (32.2–55.8)
Female sex	11,955	6,128 (51.3%)
Household size, no. of persons	11,731	3 (2–4)
Body mass index, kg/m^2^	11,893	25.4 ± 4.6
Smoking status	11,875	—
Current smoker	—	3,119 (26.3%)
Former smoker	—	3,395 (28.6%)
Never smoker	—	5,361 (45.1%)
Current occupation	11,833	—
Employed	—	8,575 (72.5%)
Unemployed	—	1,476 (12.5%)
On parental leave	—	307 (2.6%)
Retired	—	1,475 (12.5%)
Highest education	11,663	—
Compulsory school not completed	—	37 (0.3%)
Compulsory school	—	1,215 (10.4%)
Apprenticeship diploma	—	4,466 (38.3%)
Vocational school or A-levels	—	3,953 (33.9%)
University degree	—	1,992 (17.1%)
Prior SARS-CoV-2 infection	11,955	1,652 (13.8%)
Symptomatic^a^	1,608	1,209 (75.2%)
Time since prior infection, months	1,544	4.0 (3.4–4.5)
Other pre-existing conditions	—	—
Cardiovascular disease	11,892	1,355 (11.4%)
Diabetes	11,900	320 (2.7%)
Chronic lung disease	11,837	503 (4.2%)
Cancer	11,915	385 (3.2%)
Renal disease	11,913	148 (1.2%)
Liver disease	11,898	93 (0.8%)
Intake of immunosuppressants	11,887	148 (1.2%)

IQR denotes interquartile range and SD standard deviation. Continuous characteristics are summarized as means ± SD if approximately normally distributed or as medians (IQR) if otherwise. ^a^The denominator for this variable is the number of individuals with prior SARS-CoV-2 infection (i.e., 75.2% arise from 1,209/1,608).

### Incidence of SARS-CoV-2 infection and clinical features

During the six-month follow-up (311,387 person-weeks at risk), we recorded 71 cases of incident SARS-CoV-2 infection, of which 45 (63.4%) were symptomatic. The frequencies of reported symptoms are shown in Figure S1 and most commonly included new or increased cough (n = 23, 32.4%), sore throat (n = 20, 28.2%), fever (n = 19, 26.8%), new or increased muscle pain (n = 18, 25.4%), and new or increased loss of taste (n = 17, 23.9%) or smell (n = 15, 21.1%). Median duration of disease was 10 days (IQR 7–10). One case of SARS-CoV-2 infection required hospitalization (for 9 days); none were fatal. Four study participants without SARS-CoV-2 infection died from other causes (one long-term heart failure, one car accident, one suicide, one cancer).

Of the 71 cases of SARS-CoV-2 infection, 21 (29.6%) were attributable to the Alpha variant (B.1.1.7), 20 (28.2%) to the Alpha + E484K subvariant (B.1.1.7 + E484K), and 13 (18.3%) to the Delta variant (B.1.617.2). In 17 cases (23.9%), the virus subtype was not determined. Alpha and Alpha + E484K occurred exclusively during the first half of follow-up, whereas Delta occurred exclusively during the second half of follow-up (see Figure S2).

Table 2 shows incidence rates of SARS-CoV-2 infection per 100,000 person-weeks overall and separately for different periods of follow-up. Incidence rates of SARS-CoV-2 infection were 92.3 (95% CI: 70.8–120.2) per 100,000 person-weeks at weeks one to five after the first dose and 6.4 (3.9–10.4) per 100,000 person-weeks at six or more weeks after the first dose, corresponding to an incidence rate ratio of 0.07 (0.04–0.12); this indicates a reduction in incidence rates of SARS-CoV-2 infection of 93% (88%–96%) when comparing the period from week six after first dose to end of follow-up with week one to five after the first dose. For symptomatic infection, incidence rates were 57.0 (40.8–79.8) per 100,000 person-weeks at weeks one to five after the first dose and 4.4 (2.4–7.9) per 100,000 person-weeks at six or more weeks after the first dose, corresponding to an incidence rate ratio of 0.08 (0.04–0.15); this indicates a reduction in incidence rates of SARS-CoV-2 infection of 92% (85%–96%) when comparing the period from week six after first dose to end of follow-up with week one to five after the first dose.

**Table 2:** Incidence of SARS-CoV-2 infections in participants of the REDUCE study (n = 11,955)

	No. of cases	Incidence rate per 100,000 person-weeks (95% CI)	Incidence rate ratio (95% CI)
**SARS-CoV-2 infection**			

Entire follow-up	71	22.8 (18.1–28.8)	—
Week 1 to week 5 after first dose	55	92.3 (70.8–120.2)	[Reference]
Week 6 after first dose to end of follow-up	16	6.4 (3.9–10.4)	0.07 (0.04–0.12)
Week 6 to week 16 after first dose	3	2.3 (0.7–7.1)	0.02 (0.01–0.08)
Week 17 after first dose to end of follow-up	13	10.7 (6.2–18.4)	0.12 (0.06–0.21)

**Symptomatic SARS-CoV-2 infection**			

Entire follow-up	45	14.5 (10.8–19.4)	—
Week 1 to week 5 after first dose	34	57.0 (40.8–79.8)	[Reference]
Week 6 after first dose to end of follow-up	11	4.4 (2.4–7.9)	0.08 (0.04–0.15)
Week 6 to week 16 after first dose	0	No cases	No cases
Week 17 after first dose to end of follow-up	11	9.0 (5.0–16.3)	0.16 (0.08–0.31)

CI denotes confidence interval. Incidence rate ratios and 95% confidence intervals were estimated using negative binomial regression, with the period from week 1 to week 5 after first dose being used as reference.

### Effectiveness of the ultra-rapid rollout vaccination campaign

To estimate the effectiveness of the ultra-rapid rollout vaccination campaign, we compared REDUCE study participants and the whole population of the district of Schwaz (n = 84,456) with other districts of Tyrol (n = 675,649). Although the residents in the district of Schwaz and in other districts of Tyrol had a comparable age and sex structure (see Figure S3), the REDUCE study included a higher proportion of younger individuals (apart from those aged <18 years not included by design). Before the start of the vaccination campaign, 8,197 residents in the district of Schwaz (9.7%) and 41,358 residents in other districts of Tyrol (6.1%) had already had a documented SARS-CoV-2 infection.

**Figure 2:**
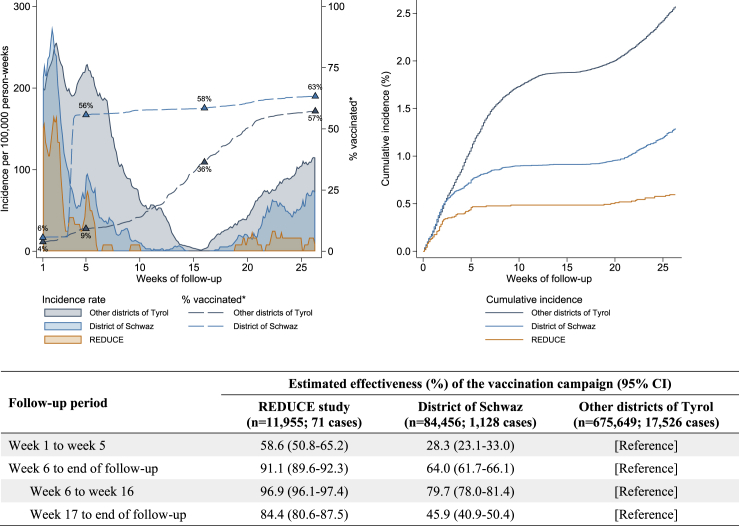
Comparison of SARS-CoV-2 incidence in the REDUCE study population and the district of Schwaz with other districts of Tyrol CI denotes confidence interval. Incidence rates in the top left panel are rolling incidence rates calculated for each day and the six preceding days. ∗Vaccinated is defined as having received two doses of the BNT162b2, mRNA-1273, or ChAdOx1 vaccine or as having received one dose of the Ad26.CoV2.S vaccine.

Figure 2 shows vaccination coverages, incidence rates, and cumulative incidences in the three groups. The percentage of the total population classified as vaccinated with two doses increased sharply in the district of Schwaz from 6% to 56% at the time of the second dose of the vaccination campaign and further to 63% by the end of follow-up. In contrast, vaccination in other districts of Tyrol progressed at a slower pace from 4% to 55% over a period of 22 weeks (i.e., until mid-August) and plateaued ∼6% points lower than in the district of Schwaz. Estimated effectiveness of the vaccination campaign in reducing incident SARS-CoV-2 infections among REDUCE participants compared with other districts of Tyrol was 58.6% (50.8%–65.2%) at weeks one to five after the first dose and 91.1% (89.6%–92.3%) at six or more weeks after the first dose (see Figure 2). The corresponding effectiveness estimates for the district of Schwaz were 28.3% (23.1%–33.0%) at weeks one to five after the first dose and 64.0% (61.7%–66.1%) at six or more weeks after the first dose compared with other districts of Tyrol. Sensitivity analyses using neighboring districts as reference yielded similar results and are shown in Figure S4. A post-hoc analysis estimating effectiveness using different cutoffs to define the first and the second follow-up period is provided in Figure S5.

### Characteristics associated with SARS-CoV-2 infection

We assessed associations of baseline characteristics with SARS-CoV-2 infection in univariable (see Table S2) and multivariable models (see Table 3) within REDUCE participants. Detailed information on each step of the multivariable model selection is provided in Table S3. In the multivariable model, the hazard ratios for SARS-CoV-2 infection were 2.01 (95% CI: 1.16–3.50) comparing participants living in households with three or more persons with those living in households with less than three persons, 3.39 (1.17–9.77) comparing participants with diabetes with those without, 3.06 (1.39–6.74) comparing participants with chronic lung disease with those without, and 0.27 (0.08–0.85) comparing participants with prior SARS-CoV-2 infection with those without. Cumulative incidence plots are provided in Figure S6. Sensitivity analyses for variables in the multivariable model comparing the first five weeks with the remaining follow-up are provided in Table 3. Notably, the hazard ratio for SARS-CoV-2 infection per ten years younger age was 1.53 (1.00–2.33) at six or more weeks after the first dose (see Table 3 and Figure S7).

**Table 3:** Multivariable adjusted hazard ratios for incident SARS-CoV-2 infection according to baseline characteristics in study participants with complete information on the covariates (n = 11,154)

Variables in the multivariable model^a^	Hazard ratio (95% CI)		
	Entire follow-up (n = 11,154; 66 cases)	Week 1 to week 5 after first dose (n = 11,154; 52 cases)	Week 6 after first dose to end of follow-up (n = 11,045; 14 cases)
Younger age, per 10 years			
Week 1 to 5 after first dose	1.07 (0.88–1.31)	1.07 (0.87–1.31)	—
Week 6 after first dose to end of follow-up	1.55 (1.02–2.33)^b^	—	1.53 (1.00–2.33)^b^
Female sex	0.80 (0.49–1.30)	0.77 (0.45–1.34)	0.90 (0.32–2.59)
Household size (≥3 vs. fewer persons)	2.01 (1.16–3.50)^b^	2.35 (1.24–4.45)^c^	1.19 (0.39–3.61)
Current smoker	0.65 (0.35–1.23)	0.62 (0.30–1.30)	0.75 (0.20–2.78)
High education^d^	1.68 (1.00–2.82)	1.61 (0.90–2.88)	1.97 (0.60–6.47)
Prior SARS-CoV-2 infection	0.27 (0.08–0.85)^b^	0.34 (0.11–1.10)	No cases
Diabetes	3.39 (1.17–9.77)^b^	3.99 (1.36–11.71)^b^	No cases
Chronic lung disease	3.06 (1.39–6.74)^c^	3.26 (1.38–7.71)^c^	2.23 (0.29–17.12)

CI denotes confidence interval. –, not applicable.

a The multivariable model was built based on data on the entire follow-up and using a backward stepwise procedure employing prespecified selection criteria for variable removal (p ≥ 0.20) and variable re-entry (p ≤ 0.10) involving the variables age, sex, household size, overweight/obese, current smoker, high education, prior SARS-CoV-2 infection, cardiovascular disease, diabetes, chronic lung disease, cancer, renal disease, liver disease, and intake of immunosuppressants, with age and sex forced to be included in the model.

b P value < 0.05.

c P value < 0.01.

d High education was defined as vocational school, A-levels, or university diploma. The number of cases among female participants were 30 over the entire follow-up, 23 over week 1–5 after first dose, and 7 over week 6 after to first dose to end of follow-up. The corresponding number of cases were 47, 38, and 9 among households with ≥3 persons; 12, 9, and 3 among current smokers; 43, 33, and 10 among participants with high education; 3, 3, and 0 among participants with prior SARS-CoV-2 infection; 4, 4, and 0 among participants with diabetes; and 7, 6, and 1 among participants with chronic lung disease.

## Discussion

In the present study, we evaluated the impact of an ultra-rapid rollout vaccination campaign conducted in March and April 2021 in the district of Schwaz in Austria on the number of new infections with SARS-CoV-2. We observed a rapid and large reduction in SARS-CoV-2 incidence during the period from week six after the first dose of the BNT162b2 mRNA vaccine to end of follow-up compared with week one to five after the first dose, both within the study and compared with other districts not offered fast-track vaccination.

A distinguishing feature of the vaccination campaign we evaluated is that it was conducted at unprecedented pace, providing vaccination to 66.9% of the eligible population within few days. The risk reductions we observed in our study (see Figure 2) closely agree with the data of the phase II/III trial of the BNT162b2 mRNA vaccine, showing vaccine efficacies in preventing COVID-19 of 95.0% (90.3%–97.3%) through 16 weeks^2^ and 91.3% (89.0%–93.2%) through six months.^12^ Previous assessments of the impact of vaccination with BNT162b2 at the population level have focused on nationwide rollouts over longer periods; largely stem from Israel,^5^^,^^13^ the UK,^14^^,^^15^^,^^16^ Canada,^17^^,^^18^ Qatar,^19^ and the US^20^^,^^21^^,^^22^; and consistently reported effectiveness estimates of more than 80%–90%.^23^ Similar effectiveness estimates were observed in studies involving health care workers^23^ in the UK,^24^ Israel,^25^ the US,^26^^,^^27^^,^^28^ Italy,^29^^,^^30^ and France.^31^ In contrast to these previous studies that used unvaccinated individuals as a comparator, the control group in our study consisted of districts that rolled out vaccination at a slower pace. Therefore, our study provides novel insight on the impact of an accelerated vaccination rollout on the control of incident infections during a pandemic.

As expected, the effectiveness of the vaccination campaign was lower in the last four to eight weeks of follow-up; this is most likely the case because ∼55%–57% of the control group were also fully immunized at that time. Moreover, lower effectiveness of the vaccination campaign toward the end of the follow-up period may also be the result of a decreasing immune response over time.^32^^,^^33^ Decreased vaccine efficacy or effectiveness after six months have also been reported by a large-scale meta-regression analysis of 18 studies.^34^ However, although effectiveness of the vaccination campaign decreased over time, it is noteworthy that its impact is still present at a time when vaccination against COVID-19 was available to the general public in all districts of Tyrol. A potential explanation is that vaccination coverage at end of follow-up was ∼6% higher in the district of Schwaz than in other districts of Tyrol, which may be attributable to earlier vaccination of all aged ≥16 years provided by the campaign at a time of vaccine scarcity.^35^

With respect to variant types, we observed a clear separation of occurrences of Alpha versus Delta variants in spring versus summer 2021, analogous to the time trends in the frequency of different variants of concern in Austria and the European Union.^36^ There were no cases of the Beta variant, which had circulated widely in the district before the study initiation. Approximately half of infections in the first half of follow-up were with the Alpha + E484K subvariant, which circulated in February/March 2021 in the state of Oregon,^37^ in South-West England,^38^ and the district of Schwaz^9^ and has previously raised concerns regarding its potential to escape from immune control after previous infection or vaccination.^10^^,^^11^ However, our study demonstrates elimination of this subvariant similar to the Alpha variant and thereby confirms *in-vitro* data on BNT162b2-elicited neutralization of this variant subtype, albeit at lower potency.^39^^,^^40^

We also identified characteristics associated with higher or lower risk of SARS-CoV-2 infection. Participants with diabetes or chronic lung disease were at higher risk of infection during the initial five weeks after the first dose. These associations persisted in analyses restricted to symptomatic infection (data not shown), thereby limiting the likelihood of medical surveillance bias through a higher frequency of medical check-ups. Interestingly, in the period six or more weeks after the first dose, the association with chronic lung disease was no longer present and no participant with diabetes experienced an SARS-CoV-2 infection. Potential explanations for this observation could be that specific patient groups only build up high-level protection after receipt of the second dose^13^ or that these population subgroups are depleted of susceptible individuals in the early phase of follow-up. Similarly, a higher risk of infection was observed in larger households, whereas a 4-fold lower risk was observed in participants with prior SARS-CoV-2 infection. Another novel finding of our study is a shift of SARS-CoV-2 cases toward younger ages in the second half of the follow-up. Given the overwhelming and consistent data of similar effectiveness across age groups according to time since vaccination,^12^^,^^13^^,^^20^^,^^41^ potential explanations include less protection of younger individuals against Delta (as observed in New York state^42^), higher vaccination coverage among elderly, or higher level of contacts with unvaccinated individuals.

### Limitations of the study

Our study has strengths and limitations. Strengths include the unique setting to study the impact of ultra-rapid vaccination delivery to the general population, a high participation rate, and rigorous validation of clinical courses of all SARS-CoV-2 cases. Furthermore, our study had a prospective cohort design and therefore does not rely on assumptions of test-negative studies^16^^,^^17^^,^^19^ reviewed recently.^43^ Although our study covers well the age groups of 18–60 years, a limitation is that it undersampled older individuals and those with preexisting diseases.^44^ Furthermore, seasonal changes in outdoor temperature and different nonpharmaceutical interventions have an effect on SARS-CoV-2 incidence rates, but these were largely across the different districts of Tyrol compared in our study. Another limitation is that the present study was conducted during phase where the variants Alpha and Delta circulated predominantly. Hence, it is unclear how these findings are transferable to emerging SARS-CoV-2 variants such as Omicron (B.1.1.529). In addition, many countries have already started vaccinating individuals with additional vaccine doses. Further investigations are needed to investigate the impact of a rapid rollout vaccination campaign with a third dose of BNT162b2 on the incidence of SARS-CoV-2 infections. Also, given that study participants were not regularly screened for presence of SARS-CoV-2, some underascertainment of asymptomatic infections is likely, resulting in an underestimation of the number of incident SARS-CoV-2 infections. In addition, although we provide results adjusted for a variety of participant characteristics, our results may still be subject to residual confounding, as we were unable to measure workplace regulations, leisure activities, and adherence to hygiene regulations, among others. Finally, due to data protection regulations, we could not obtain contact tracing data for SARS-CoV-2 cases that would have allowed quantification of secondary attack rates among vaccinated individuals.

In conclusion, the vaccination campaign led to a rapid and large reduction of incident SARS-CoV-2 in study participants and the whole district. Our study illustrates the impact of an accelerated vaccination rollout in controlling the pandemic.

## STAR★Methods

### Key resources table

**Table:** 

REAGENT or RESOURCE	SOURCE	IDENTIFIER
**Deposited data**		

Open-source data on SARS-CoV-2 infections at district-level. Accessed October 25, 2021.	https://www.data.gv.at/katalog/dataset/4b71eb3d-7d55-4967-b80d-91a3f220b60c	N/A

**Software and algorithms**		

Stata	https://www.stata.com/	Version 15.1

**Other**		

Data obtained by questionnaire.	This paper.	N/A
Data on number of vaccinated individuals at district-level.	This paper. Obtained from Land Tirol.	N/A
Data on incident SARS-CoV-2 infections of study participants.	This paper. Obtained from Austrian Agency for Health and Food Safety.	N/A

### Resource availability

#### Lead contact

Further information and requests for resources should be directed to and will be fulfilled by the lead contact, Peter Willeit (peter.willeit@i-med.ac.at).

#### Materials availability

This study did not generate new unique reagents.

### Experimental model and subject details

#### Study oversight and reporting

The study was approved by the ethics committee of the Medical University of Innsbruck (no. 1095/2021) and registered at the Austrian Federal Office for Safety in Health Care.^45^ Results are reported in accordance with the Strengthening the Reporting of Observational studies in Epidemiology (STROBE) guidelines (see Table S4).^46^

### Method details

#### Study design and participants

The REDUCE study is an observational multicenter prospective cohort study nested within the ultra-rapid rollout vaccination campaign in the district of Schwaz, Tyrol, Austria. The campaign offered vaccination with the BNT162b2 mRNA vaccine to residents of the district within a short time span of six days per dose (first dose: 11–16 March 2021, second dose: 8–13 April 2021). After excluding 6,633 individuals already vaccinated prior to the campaign (i.e. individuals aged ≥80 years, at high risk, living in nursing homes, or working in healthcare) and 13,825 individuals aged <16 years, 63,998 of 84,456 individuals with a primary residence in the district of Schwaz were eligible for vaccination (see Figure 1). 42,807 of these individuals were vaccinated, corresponding to 66.9% of the eligible population. Vaccination was offered at 26 vaccination centers and was delivered by intramuscular injection of a 30μg dose of the BNT162b2 mRNA COVID-19 vaccine. After vaccination, individuals were asked to rest for 15–20 min or – in case of known allergies^47^ – for 30–60 min.

During the 15–20 min resting period, at nine of the 26 vaccination centers (during 12–15 March 2021), individuals were asked by trained staff whether they wished to participate in the REDUCE study. All participants provided written informed consent and completed a self-administered paper questionnaire on sociodemographic characteristics (i.e. age, sex, occupation, education, household size), lifestyle factors (i.e. smoking, height, weight), prior SARS-CoV-2 infection, and their broader medical history (i.e. cardiovascular disease, diabetes, chronic lung disease, cancer, renal disease, liver disease, and intake of immunosuppressants). In case participants required support while completing the questionnaire, they could contact on-site staff for assistance. Individuals were eligible for inclusion if they received vaccination as part of the campaign, were aged ≥18 years, and were legally competent. We excluded individuals who did not fulfil the criteria for vaccination as per European Medicines Agency (EMA) approval,^48^ including those given the seventh dose of a BNT162b2 vial (i.e. the extra dose often extractable from a six-dose vial with low dead-volume syringes). 11,955 of eligible 17,639 individuals at the nine vaccination centers were included in the study, corresponding to a participation rate of 67.8% (see Figure 1).

#### Outcomes definition and ascertainment

The primary outcome was incidence of SARS-CoV-2 infection confirmed by RT-PCR testing. Secondary outcomes were (i) symptomatic SARS-CoV-2 infection defined as in the BNT162b2 phase II/III trial^2^ as having one or more symptoms (including fever ≥38°C, new or increased cough, new or increased shortness of breath, chills, new or increased muscle pain, new or increased loss of smell or taste, sore throat, diarrhea, vomiting, or pneumonia), (ii) hospitalization due to SARS-CoV-2 infection, and (iii) death due to SARS-CoV-2 infection defined as death with SARS-CoV-2 as the underlying cause or any death within 21 days of infection onset. Incident outcomes were identified by the Austrian Agency for Health and Food Safety, which hosts a central database of all documented SARS-CoV-2 infections in Austria, including virus sequencing data. Information on clinical courses of infections, including symptoms and need for hospitalization, was obtained by local health authorities through routine structured telephone interviews at time of diagnosis and validated at end of follow-up. Causes of death were adjudicated by review of death certificates and by consulting medical examiners or coroners.

#### Definition of follow-up periods

For each participant, we recorded incident SARS-CoV-2 infections from the date of receipt of the first BNT162b2 dose (i.e., a date between 12 and 15 March 2021) over a follow-up of up to six months. Time-to-event data were censored at time of SARS-CoV-2 diagnosis, death, withdrawal of consent, or end of follow-up, whichever was first. Additionally, we censored individuals after five weeks of follow-up in case they deviated from the vaccination regimen recommended by Austria’s national vaccination committee, i.e. (i) they did not receive a second dose of BNT162b2 within 19–42 days after the first dose and (ii) they did not have a positive history of SARS-CoV-2 infection five weeks after receiving the first dose. We assessed incidence over the entire follow-up and separately for the following follow-up periods: (i) week one to week five after first dose; (ii) week six to week 16 after first dose; and (iii) week 17 after first dose to end of follow-up. We pre-specified to split the follow-up periods at week five to facilitate comparisons to the BNT162b2 phase II/III trial^2^ and at week 16 to investigate the stability of effectiveness estimates over time.

### Quantification and statistical analysis

To determine the target sample size of our study, we modelled incidence rates of SARS-CoV-2 infection assuming (i) a weekly incidence rate of 100 per 100,000 individuals during initial follow-up, (ii) a 95% relative risk reduction achieved five weeks after the first dose, and (iii) a drop-out rate of 5%. We estimated 95% CIs of the incidence rates using the Stata function stptime, which relies on the quadratic approximation to the Poisson log likelihood for the log-rate parameter. A sample size of at least 5,000 participants was deemed to provide an acceptably narrow 95% CI of the SARS-CoV-2 incidence rate.

In the primary analysis, we calculated incidence rates and 95% CIs for SARS-CoV-2 infection per 100,000 person-weeks separately for the different follow-up periods, and compared them by estimating incidence rate ratios using negative binomial regression.

To assess the impact of the vaccination campaign, we compared incidence rates of SARS-CoV-2 infection in the REDUCE study and the whole district of Schwaz with all other districts of the Federal State of Tyrol that vaccinated residents at a slower pace and in a staggered manner (with vaccination available to individuals aged ≥16 years from 28 May 2021 onwards). By combining our study data with publicly available data,^7^ we calculated cumulative incidence curves and effectiveness estimates (1-hazard ratios) based on probabilities of remaining event-free at each of the days during the course of the REDUCE study. Specifically, we used data of the Austrian epidemiological reporting system^7^ which contains reported SARS-CoV-2 infections and deaths for each day since February 2020. Based on these data, we estimated hazard ratios and corresponding effectiveness estimates as described elsewhere.^49^

To identify baseline characteristics associated with incident SARS-CoV-2 infection, we estimated hazard ratios and 95% CIs using univariable and multivariable Cox regression models. The multivariable model was built using a backward stepwise procedure employing pre-specified selection criteria for variable removal (p ≥ 0.20) and variable re-entry (p ≤ 0.10), considering the variables age, sex, household size, overweight/obese, current smoker, high education, prior SARS-CoV-2 infection, cardiovascular disease, diabetes, chronic lung disease, cancer, renal disease, liver disease, and intake of immunosuppressants, with age and sex forced to be included in the model. Because the proportional hazards assumption was violated for age (see Table S5), an interaction term between follow-up (split at end of week five) and age was included in the model. We applied complete case analysis, since there were only a few missing values (<2%) in the variables. Sensitivity analyses of the multivariable model involved interaction terms between follow-up and all other variables in the model. Analyses were carried out with Stata 15.1 using two-sided p-values and 95% CIs.

## Data Availability

• This paper analyzes existing, publicly available data. The websites where these datasets can be accessed are listed in the key resources table. In addition, the paper analyzes participant-level data, which cannot be deposited in a public repository because of data protection regulations. • This paper does not report original code. • Any additional information required to reanalyze the data reported in this paper is available from the lead contact upon request.
