# Supplementary material for: Ultra-rapid rollout vaccination with BNT162b2 to reduce SARS-CoV-2 infections in the general population
Source: iScience. 2022 Nov 7;25(11):105380. doi: 10.1016/j.isci.2022.105380 (PMC9639213; doi:10.1016/j.isci.2022.105380)

iScience, Volume *25*

Supplemental information

Ultra-rapid rollout vaccination with BNT162b2 to reduce SARS-CoV-2 infections in the general population

Lena Tschiderer, Lisa Seekircher, Lukas Richter, Dorothee von Laer, Cornelia Lass-Flörl, Lukas Forer, Sebastian Schönherr, Florian Krammer, Sabine Embacher-Aichhorn, Herbert Tilg, Günter Weiss, Franz Allerberger, and Peter Willeit

SUPPLEMENTARY TABLES

Table S1. Baseline characteristics according to adherence to the vaccination regimen as recommended by the national vaccination committee, related to Table 1.

Characteristic	Adherence to the vaccination regimen recommended by the national vaccination committee No. (%), mean ± SD, or median (IQR)		P-value[a]
	Adhered (n=11,893)	Deviated (n=62)	
Age, years	44.6 (32.2-55.8)	43.5 (34.4-55.2)	0.985
Female sex	6,092 (51.2%)	36 (58.1%)	0.282
Household size, no. of persons	3 (2-4)	3 (2-4)	0.867
Body mass index, kg/m^2	25.4 ± 4.6	25.8 ± 9.7	0.536
Smoking status			0.173
Current smoker	3,101 (26.3%)	18 (29.0%)	
Former smoker	3,372 (28.5%)	23 (37.1%)	
Never smoker	5,340 (45.2%)	21 (33.9%)	
Current occupation			0.048
Employed	8,539 (72.5%)	36 (60.0%)	
Unemployed	1,464 (12.4%)	12 (20.0%)	
On parental leave	303 (2.6%)	4 (6.7%)	
Retired	1,467 (12.5%)	8 (13.3%)	
Highest education			0.819
Compulsory school not completed	37 (0.3%)	0 (0.0%)	
Compulsory school	1,209 (10.4%)	6 (10.0%)	
Apprenticeship diploma	4,447 (38.3%)	19 (31.7%)	
Vocational school or A-levels	3,930 (33.9%)	23 (38.3%)	
University degree	1,980 (17.1%)	12 (20.0%)	
Other pre-existing conditions			
Cardiovascular disease	1,348 (11.4%)	7 (11.3%)	0.979
Diabetes	317 (2.7%)	3 (5.0%)	0.218
Chronic lung disease	497 (4.2%)	6 (9.7%)	0.047
Cancer	381 (3.2%)	4 (6.5%)	0.140
Renal disease	145 (1.2%)	3 (4.8%)	0.042
Liver disease	92 (0.8%)	1 (1.6%)	0.386
Intake of immunosuppressants	145 (1.2%)	3 (4.8%)	0.042

IQR denotes interquartile range and SD standard deviation. Continuous characteristics are summarized as means ± SD if approximately normally distributed or as medians (IQR) if otherwise. [a]P-value obtained from t-test for approximately normally distributed continuous variables, Mann-Whitney U test for non-normally distributed continuous variables, χ^2-test for categorical variables with expected frequencies ≥5 in at least 80% of the cells of the cross-table, and Fisher's exact test, otherwise.

Table S2. Unadjusted hazard ratios for incident SARS-CoV-2 infection according to baseline characteristics in 11,154 participants of the REDUCE study with complete information on the covariates, related to Table 3.

	Incident infection (n=66)	No infection (n=11,088)	Unadjusted hazard ratio (95% CI)	P-value
Younger age, per 10 years				
Week 1 to week 5 after first dose	44.8 (30.6-53.5)	43.9 (31.8-55.3)	1.09 (0.90-1.31)	0.375
Week 6 after first dose to end of follow-up	30.2 (24.8-42.6)	43.9 (31.8-55.3)	1.57 (1.04-2.36)	0.031
Female sex	30 (45.5%)	5,676 (51.2%)	0.80 (0.49-1.29)	0.354
Household size (≥3 vs. fewer persons)	47 (71.2%)	6,039 (54.5%)	2.06 (1.21-3.52)	0.008
Overweight/obese[a]	33 (50.0%)	5,139 (46.3%)	1.16 (0.71-1.87)	0.554
Current smoker	12 (18.2%)	2,883 (26.0%)	0.63 (0.34-1.18)	0.152
Currently employed	53 (80.3%)	8,141 (73.4%)	1.47 (0.80-2.70)	0.211
High education[b]	43 (65.2%)	5,693 (51.3%)	1.77 (1.07-2.93)	0.027
Prior SARS-CoV-2 infection	3 (4.5%)	1,553 (14.0%)	0.29 (0.09-0.93)	0.038
Other pre-existing conditions				
Cardiovascular disease	7 (10.6%)	1,208 (10.9%)	0.97 (0.44-2.12)	0.938
Diabetes	4 (6.1%)	272 (2.5%)	2.56 (0.93-7.02)	0.069
Chronic lung disease	7 (10.6%)	456 (4.1%)	2.75 (1.26-6.03)	0.011
Cancer	1 (1.5%)	331 (3.0%)	0.50 (0.07-3.61)	0.493
Renal disease	0 (0.0%)	124 (1.1%)	-	-
Liver disease	1 (1.5%)	78 (0.7%)	2.15 (0.30-15.49)	0.448
Intake of immunosuppressants	0 (0.0%)	131 (1.2%)	-	-

CI denotes confidence interval. [a]Overweight/obese was defined as a body mass index of 25 kg/m² or higher. [b]High education was defined as vocational school, A-levels, or university diploma.

Table S3. P-values of variables for each step of the multivariable model selection procedure, related to Table 3.

Variable	P-value[a]							
	Step 1 LL -593.8266	Step 2 LL -594.509	Step 3 LL -595.339	Step 4 LL -595.343	Step 5 LL -595.500	Step 6 LL -595.748	Step 7 LL -596.085	Step 8 LL -596.655
Age	0.590	0.576	0.565	0.568	0.528	0.545	0.622	0.507
Interaction of age and time	0.097	0.097	0.098	0.098	0.098	0.098	0.100	0.109
Female sex	0.617	0.607	0.604	0.604	0.590	0.590	0.480	0.366
Household size (≥3 vs. fewer persons)	0.015	0.014	0.014	0.014	0.014	0.014	0.014	0.013
Overweight/obese[b]	0.417	0.412	0.419	0.412	0.415	0.412	–	–
Current smoker	0.177	0.177	0.176	0.175	0.177	0.178	0.181	0.189
Currently employed	0.330	0.315	0.303	0.305	0.296	0.305	0.300	–
High education[c]	0.052	0.050	0.051	0.051	0.050	0.050	0.058	0.052
Prior SARS-CoV-2 infection	0.022	0.023	0.023	0.023	0.024	0.024	0.024	0.025
Cardiovascular disease	0.854	0.873	0.934	–	–	–	–	–
Diabetes	0.027	0.027	0.030	0.026	0.025	0.023	0.018	0.024
Chronic lung disease	0.004	0.005	0.005	0.005	0.005	0.005	0.005	0.006
Cancer	0.616	0.613	0.605	0.606	–	–	–	–
Renal disease	1.000	1.000	–	–	–	–	–	–
Liver disease	0.357	0.356	0.418	0.417	0.427	–	–	–
Intake of immunosuppressants	1.000	–	–	–	–	–	–	–

LL denotes log likelihood; –, variable removed from model due to selection procedure. [a]The multivariable model was built based on data on the entire follow-up and using a backward stepwise procedure employing pre-specified selection criteria for variable removal (P≥0.20) and variable re-entry (P≤0.10). No variable reached the significance threshold to re-enter the model after it was removed from the model. [b]Overweight/obese was defined as a body mass index of 25 kg/m² or higher. [c]High education was defined as vocational school, A-levels, or university diploma.

Table S4. STROBE checklist, related to STAR Methods.

	Item No	Recommendation	Page No
Title and abstract	1	(*a*) Indicate the study's design with a commonly used term in the title or the abstract	1
		(*b*) Provide in the abstract an informative and balanced summary of what was done and what was found	2
**Introduction**			
Background/ rationale	2	Explain the scientific background and rationale for the investigation being reported	3
Objectives	3	State specific objectives, including any prespecified hypotheses	3
**Methods**			
Study design	4	Present key elements of study design early in the paper	18
Setting	5	Describe the setting, locations, and relevant dates, including periods of recruitment, exposure, follow-up, and data collection	18
Participants	6	(*a*) Give the eligibility criteria, and the sources and methods of selection of participants. Describe methods of follow-up	18-19
		(*b*) For matched studies, give matching criteria and number of exposed and unexposed	NA
Variables	7	Clearly define all outcomes, exposures, predictors, potential confounders, and effect modifiers. Give diagnostic criteria, if applicable	18-19
Data sources/ measurement	8*	For each variable of interest, give sources of data and details of methods of assessment (measurement). Describe comparability of assessment methods if there is more than one group	18-19
Bias	9	Describe any efforts to address potential sources of bias	18-21
Study size	10	Explain how the study size was arrived at	20
Quantitative variables	11	Explain how quantitative variables were handled in the analyses. If applicable, describe which groupings were chosen and why	20-21
Statistical methods	12	(*a*) Describe all statistical methods, including those used to control for confounding	20-21
		(*b*) Describe any methods used to examine subgroups and interactions	20-21
		(*c*) Explain how missing data were addressed	20-21<br>Tab 3<br>Tab S2
		(*d*) If applicable, explain how loss to follow-up was addressed	19-21
		(*e*) Describe any sensitivity analyses	20-21
**Results**			
Participants	13*	(a) Report numbers of individuals at each stage of study—eg numbers potentially eligible, examined for eligibility, confirmed eligible, included in the study, completing follow-up, and analyzed	18
		(b) Give reasons for non-participation at each stage	18
		(c) Consider use of a flow diagram	Fig 2
Descriptive data	14*	(a) Give characteristics of study participants (eg demographic, clinical, social) and information on exposures and potential confounders	4, Tab 1
		(b) Indicate number of participants with missing data for each variable of interest	Tab 1
		(c) Summarize follow-up time (eg, average and total amount)	4
Outcome data	15*	Report numbers of outcome events or summary measures over time	4-6
Main results	16	(a) Give unadjusted estimates and, if applicable, confounder-adjusted estimates and their precision (eg, 95% confidence interval). Make clear which confounders were adjusted for and why they were included	4-6
		(b) Report category boundaries when continuous variables were categorized	4-6
		(c) If relevant, consider translating estimates of relative risk into absolute risk for a meaningful time period	NA

Other analyses	17	Report other analyses done—eg analyses of subgroups and interactions, and sensitivity analyses	5-6
**Discussion**			
Key results	18	Summarize key results with reference to study objectives	7
Limitations	19	Discuss limitations of the study, taking into account sources of potential bias or imprecision. Discuss both direction and magnitude of any potential bias	9
Interpretation	20	Give a cautious overall interpretation of results considering objectives, limitations, multiplicity of analyses, results from similar studies, and other relevant evidence	7-10
Generalizability	21	Discuss the generalizability (external validity) of the study results	7-10
**Other information**			
Funding	22	Give the source of funding and the role of the funders for the present study and, if applicable, for the original study on which the present article is based	10

Table S5. Investigation of the proportional hazards assumption in Cox regression, related to STAR Methods.

Variable	Schoenfeld residuals				Log-log plot of survival	Kaplan-Meier versus predicted survival plot
	ρ	X_1^2	P-value	Conclusion		
Younger age, per 10 years	0.3081	6.034	0.014	×	×	×
Female sex	0.1125	0.835	0.361	✓	✓	✓
Household size (≥3 vs. fewer persons)	-0.0233	0.036	0.850	✓	✓	✓
Overweight/obese[a]	-0.1776	2.083	0.149	✓	✓	✓
Current smoker	0.0318	0.067	0.796	✓	✓	✓
Currently employed	0.0191	0.024	0.877	✓	✓	✓
High education[b]	0.1510	1.505	0.220	✓	✓	✓
Prior SARS-CoV-2 infection	-0.1048	0.725	0.395	✓	✓	✓
Other pre-existing conditions						
Cardiovascular disease	-0.0017	<0.001	0.989	✓	✓	✓
Diabetes	-0.0851	0.478	0.489	✓	✓	✓
Chronic lung disease	0.0069	0.003	0.955	✓	✓	✓
Cancer	-0.0806	0.429	0.512	✓	✓	✓
Renal disease	–	–	–	–	–	–
Liver disease	0.2820	5.248	0.022	×	✓	✓
Intake of immunosuppressants	–	–	–	–	–	–

Results presented in this table are for the univariable model. For the log-log plot and the plot on Kaplan-Meier versus predicted survival, we compared the age groups 18-29, 30-44, and ≥45 years. ✓, consistent with proportional hazards. ×, not consistent with proportional hazards. –, not applicable (no incident infection in individuals with renal disease and intake of immunosuppressants). [a]Overweight/obese was defined as a body mass index of 25 kg/m² or higher. [b]High education was defined as vocational school, A-levels, or university diploma.

SUPPLEMENTARY FIGURES

Figure S1. Symptoms reported by cases of SARS-CoV-2 infection, related to Table 2.

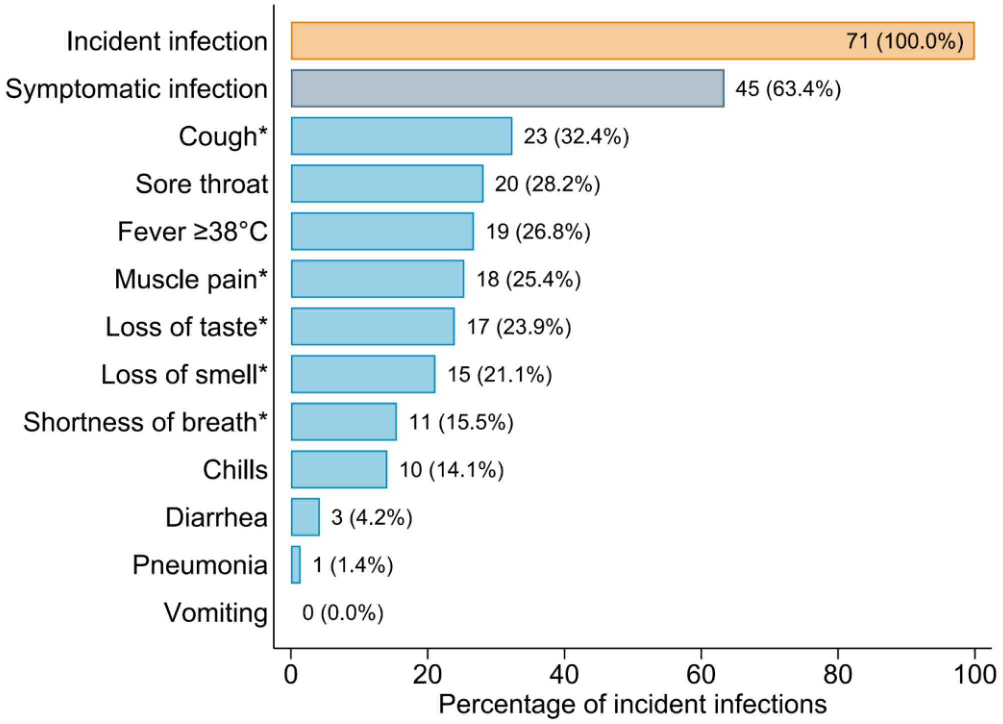

*Restricted to new occurrence or worsening of the symptom during SARS-CoV-2 infection.

Figure S2. Number of cases of SARS-CoV-2 infection by type of variant, related to Table 2.

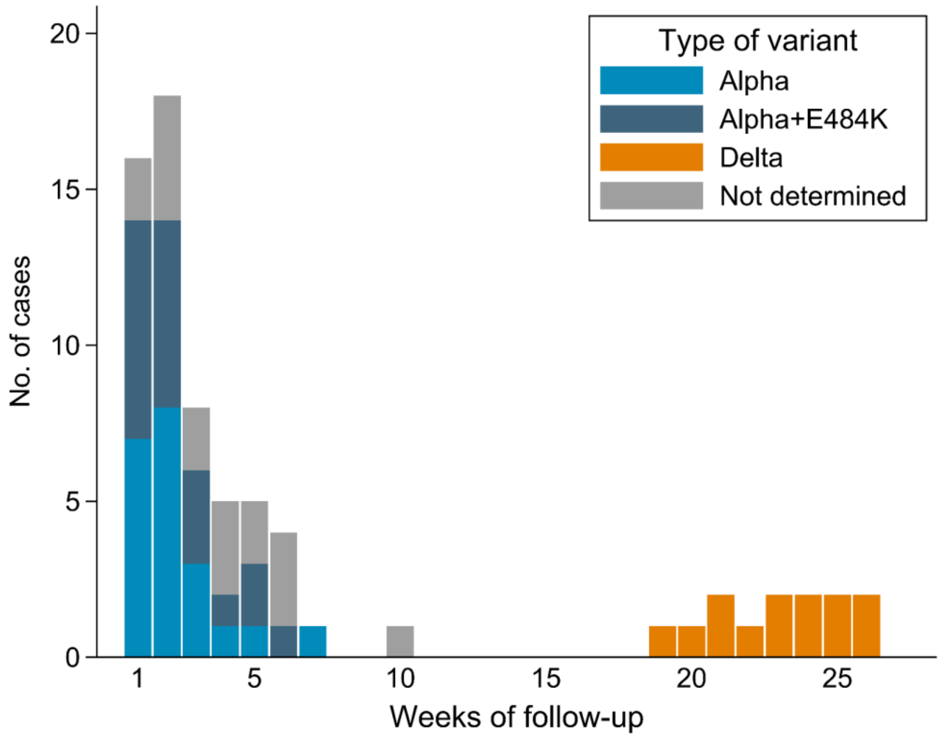

Figure S3. Age and sex distribution of individuals in the REDUCE study population, in the district of Schwaz, and in other districts in Tyrol, related to Figure 1.

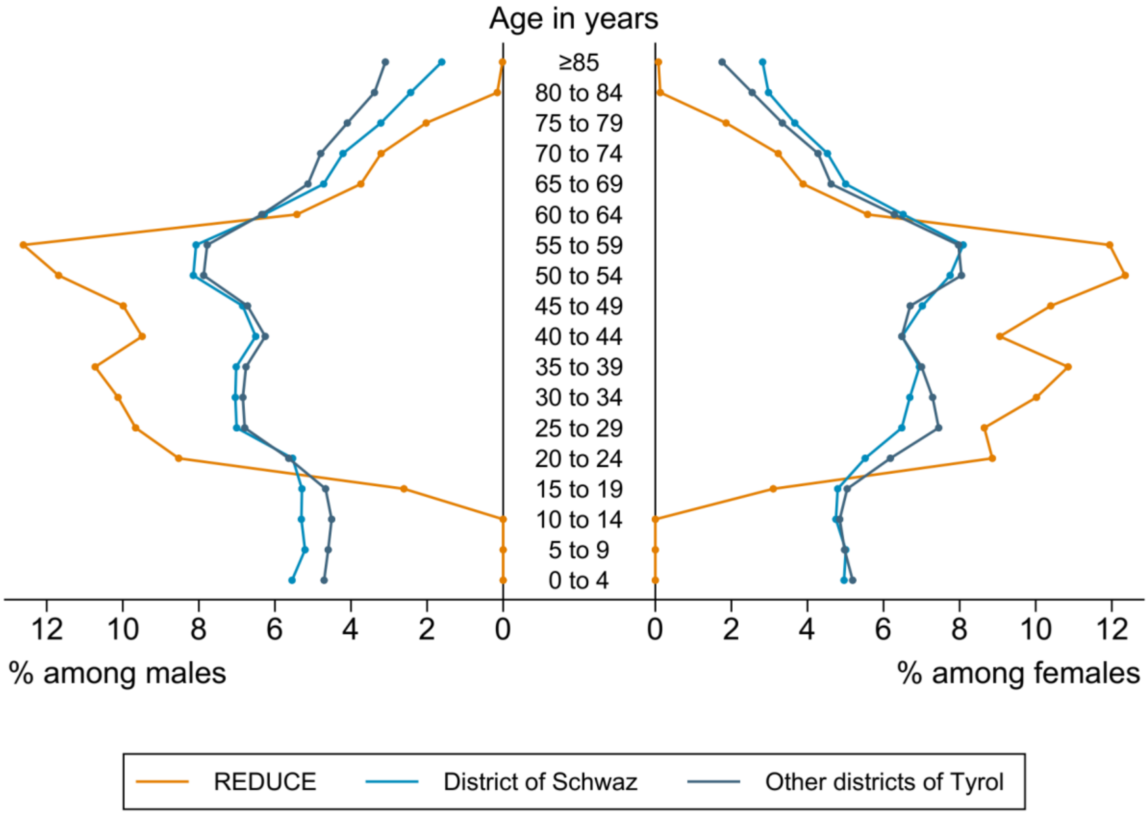

Data on age and sex distributions in the district of Schwaz and other districts of Tyrol are from 1 January 2021 and are publicly available at the Statistik Austria webpage.

Figure S4. Sensitivity analysis using the neighboring districts of Schwaz (Innsbruck-Land and Kufstein) as the reference group to estimate the effectiveness of the ultra-rapid rollout vaccination campaign, related to Figure 1.

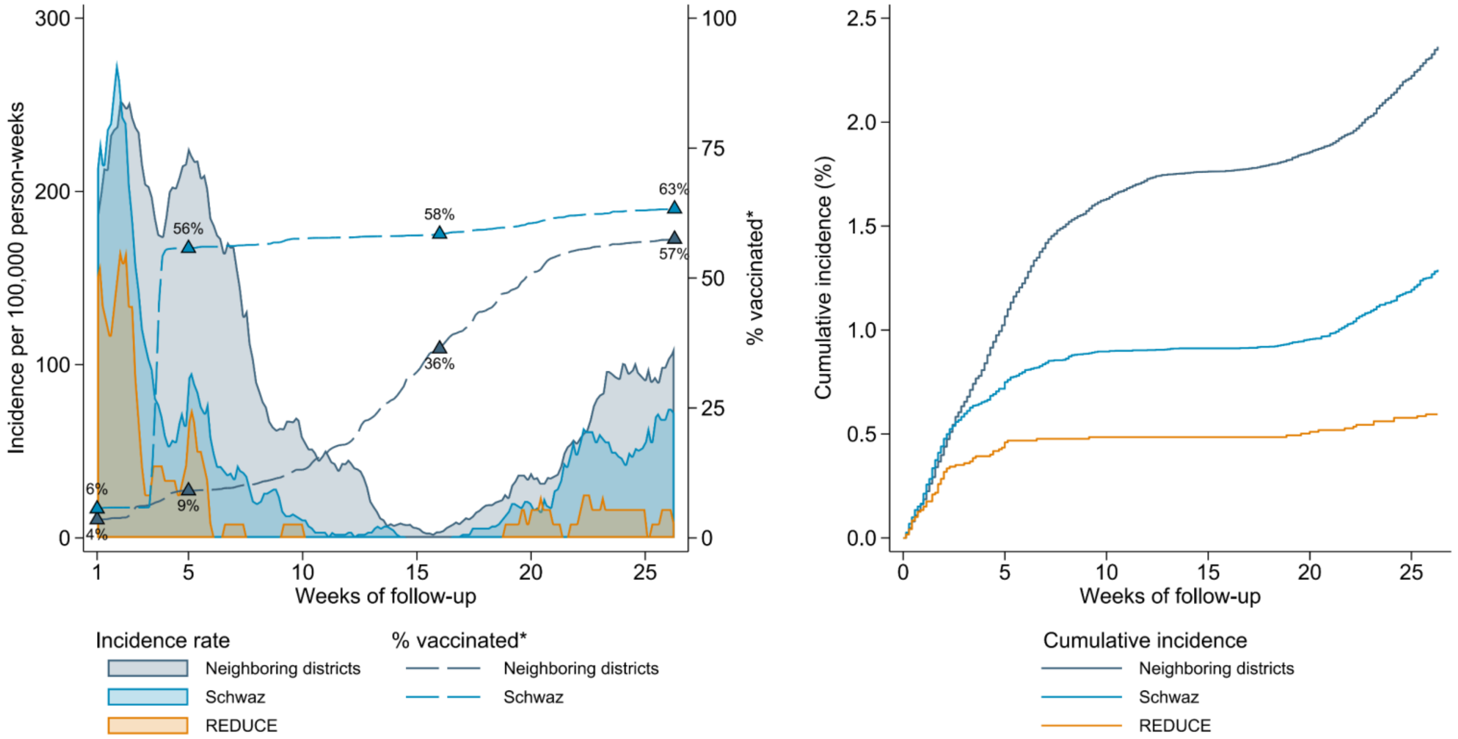

Follow-up period	Estimated effectiveness (%) of the vaccination campaign (95% CI)		
	**REDUCE study (n=11,955; 71 cases)**	**District of Schwaz (n=84,456; 1,128 cases)**	**Neighboring districts (n=292,778; 6,977 cases)**
Week 1 to week 5	57.8 (49.6-64.6)	26.8 (21.1-32.1)	[Reference]
Week 6 to end of follow-up	89.7 (87.9-91.3)	58.7 (55.6-61.5)	[Reference]
Week 6 to week 16	96.4 (95.5-97.1)	76.9 (74.5-79.1)	[Reference]
Week 17 to end of follow-up	82.0 (77.1-85.8)	37.3 (30.5-43.4)	[Reference]

CI denotes confidence interval. Incidence rates in the top left panel are rolling incidence rates calculated for each day and the six preceding days. *Vaccinated is defined as having received two doses of the BNT162b2, mRNA-1273, or ChAdOx1 vaccine or as having received one dose of the Ad26.CoV2.S vaccine.

Figure S5. Effectiveness estimates of the vaccination campaign according to different cut-offs for definition the follow-up periods, related to Figure 1.

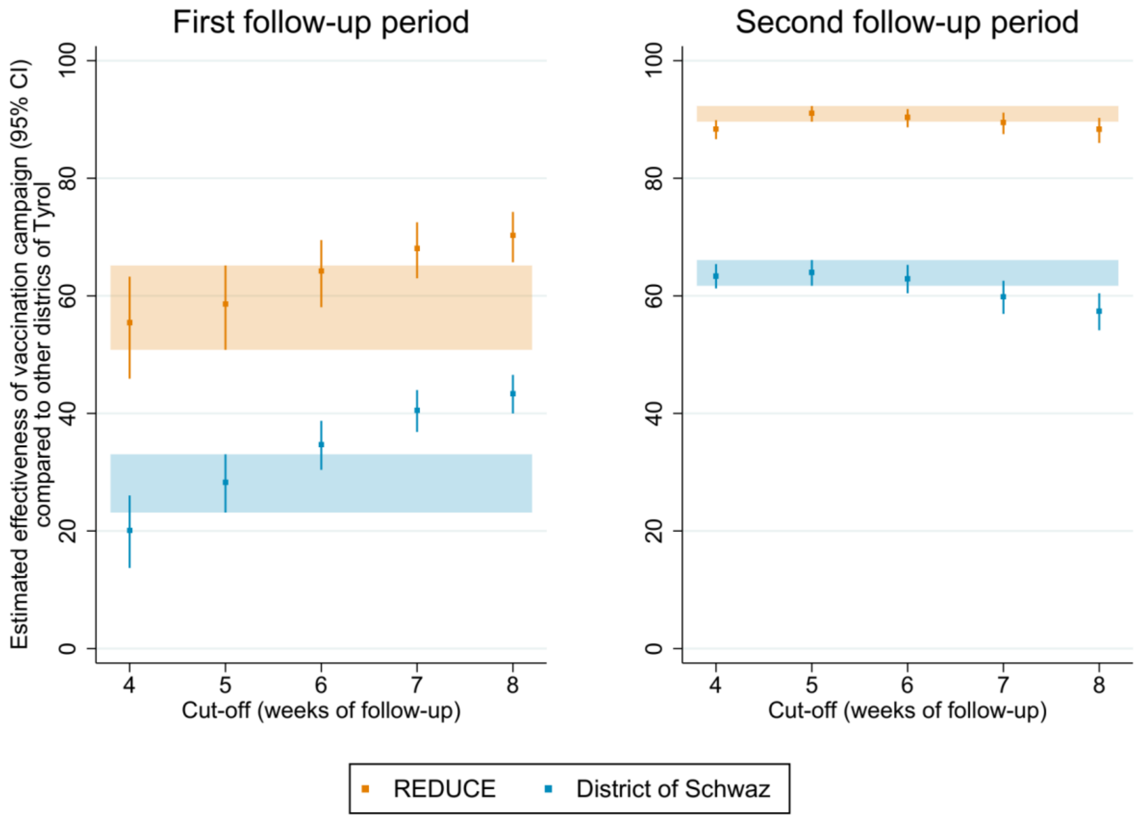

CI denotes confidence interval. The orange and blue ribbons depict lower and upper bounds of 95% confidence intervals for effectiveness estimates based on the cut-off we selected for our primary analysis (i.e., after 5 weeks of follow-up).

Figure S6. Cumulative incidence of SARS-CoV-2 infection in the REDUCE study split by household size, prior SARS-CoV-2 infection, history of diabetes, and chronic lung disease, related to Table 3.

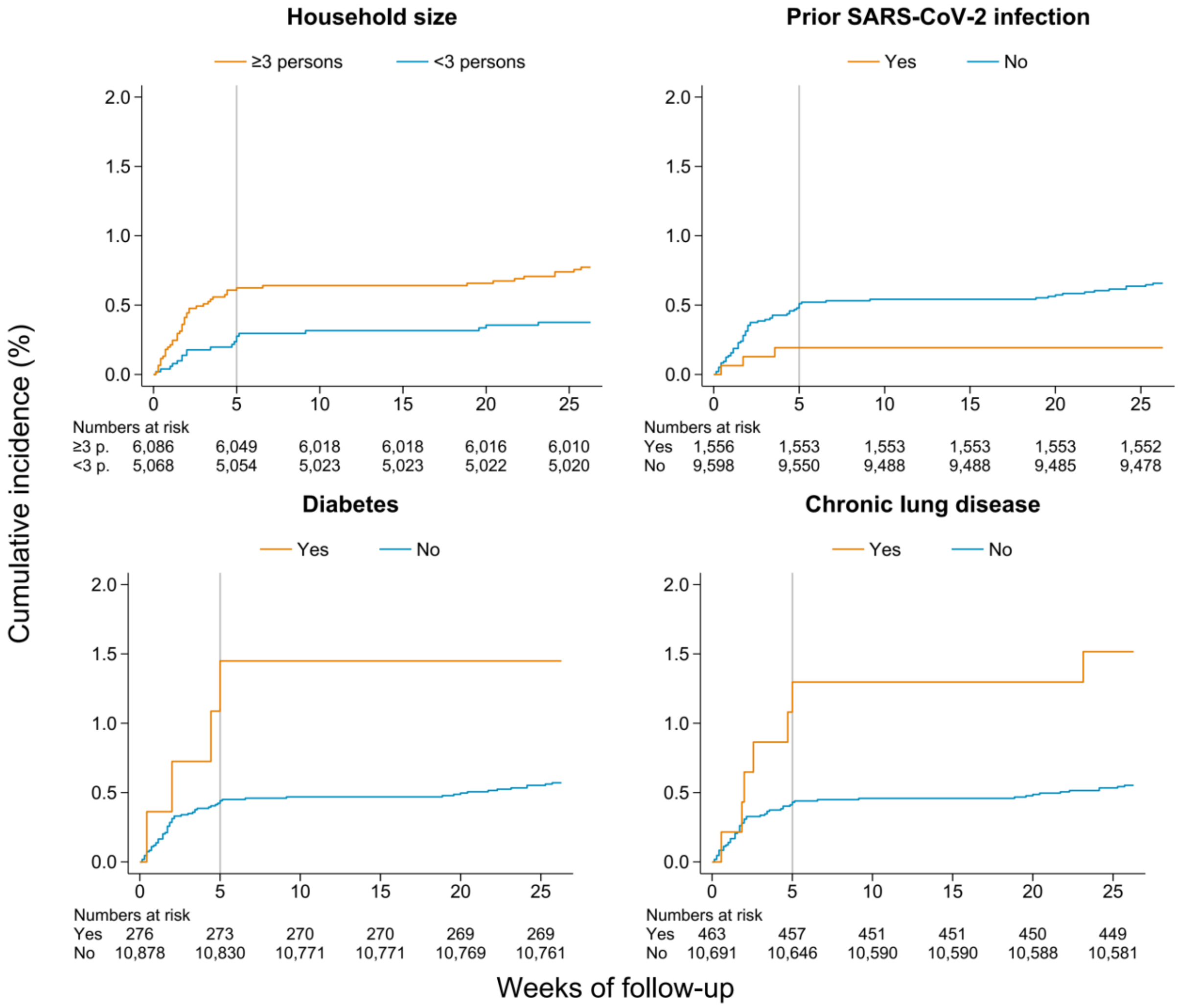

Figure S7. Age of the cases with incident SARS-CoV-2 infection stratified by variant plotted against time from baseline to diagnosis, related to Table 3.

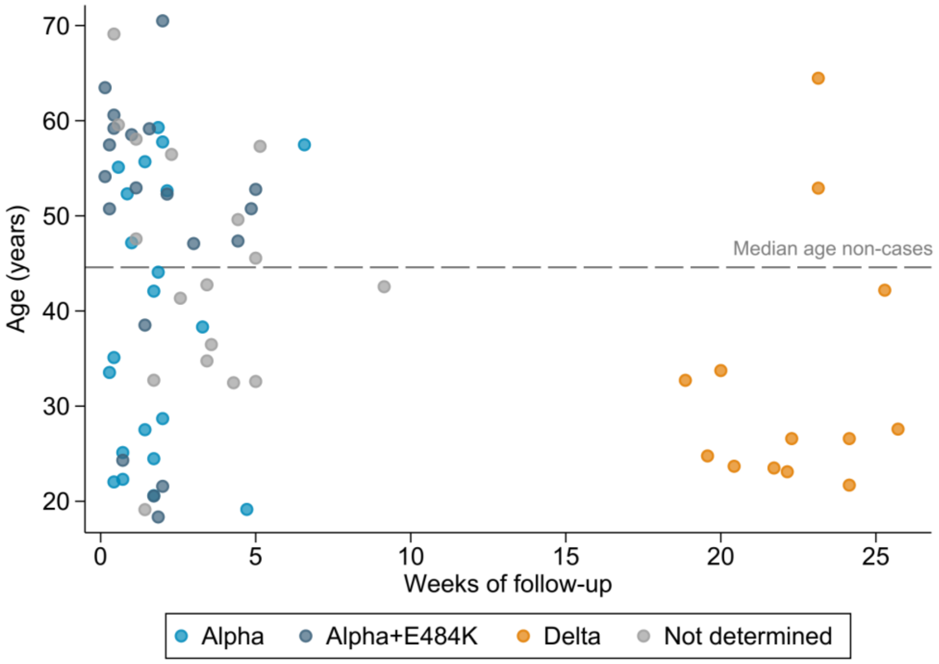

Supplement: Document S1. Figures S1–S7 and Tables S1–S5 [file mmc1.pdf]
